# Evidence for Thalamic Involvement in the Thermal Grill Illusion: An fMRI Study

**DOI:** 10.1371/journal.pone.0027075

**Published:** 2011-11-11

**Authors:** Fredrik Lindstedt, Bo Johansson, Sofia Martinsen, Eva Kosek, Peter Fransson, Martin Ingvar

**Affiliations:** 1 Department of Clinical Neuroscience, Osher Center for Integrative Medicine, Stockholm Brain Institute, Karolinska Institutet, Stockholm, Sweden; 2 Somedic AB, Hörby, Sweden; Hangzhou Normal University, China

## Abstract

**Background:**

Perceptual illusions play an important role in untangling neural mechanisms underlying conscious phenomena. The thermal grill illusion (TGI) has been suggested as a promising model for exploring percepts involved in neuropathic pain, such as cold-allodynia (pain arising from contact with innocuous cold). The TGI is an unpleasant/painful sensation from touching juxtapositioned bars of cold and warm innocuous temperatures.

**Aim:**

To develop an MRI-compatible TGI-unit and explore the supraspinal correlates of the illusion, using fMRI, in a group of healthy volunteers.

**Methods:**

We constructed a TGI-thermode allowing the rapid presentation of warm(41°C), cold(18°C) and interleaved(41°C+18°C = TGI) temperatures in an fMRI-environment. Twenty volunteers were tested. The affective-motivational (“unpleasantness”) and sensory-disciminatory (“pain-intensity”) dimensions of each respective stimulus were rated. Functional images were analyzed at a corrected α-level <0.05.

**Results:**

The TGI was rated as significantly more unpleasant and painful than stimulation with each of its constituent temperatures. Also, the TGI was rated as significantly more unpleasant than painful. Thermal stimulation versus neutral baseline revealed bilateral activations of the anterior insulae and fronto-parietal regions. Unlike its constituent temperatures the TGI displayed a strong activation of the right (contralateral) thalamus. Exploratory contrasts at a slightly more liberal threshold-level also revealed a TGI-activation of the right mid/anterior insula, correlating with ratings of unpleasantness(rho = 0.31).

**Conclusion/Significance:**

To the best of our knowledge, this is the first fMRI-study of the TGI. The activation of the anterior insula is consistent with this region's putative role in processing of homeostatically relevant feeling-states. Our results constitute the first neurophysiologic evidence of thalamic involvement in the TGI. Similar thalamic activity has previously been observed during evoked cold-allodynia in patients with central neuropathic pain. Our results further the understanding of the supraspinal correlates of the TGI-phenomenon and pave the way for future inquiries into if and how it may relate to neuropathic pain.

## Introduction

Illusions in the visual and somatosensory domain have contributed considerably to our understanding of the neural mechanisms involved in various conscious processes. Perceptual illusions allow the testing of models for conscious phenomena and – importantly – when coupled to neurophysiological measurements, inferences about the underlying neural substrates. One such potentially useful sensory illusion is the thermal grill illusion (TGI). The TGI was first described by Torsten Thunberg in 1898 [1]. It is as an unpleasant, potentially painful, burning sensation that arises when touching an alternating pattern of innocuous cold and warm temperatures. The quality of TGI phenomenon is related to the burning of cold-pain [2] as well as the paradoxical heat that can be felt during dynamic cooling of the skin [3], [4]. Importantly, the TGI uses innocuous temperatures to evoke such sensory manifestations usually attributed to noxious modalities.

The thermal grill has been suggested by Craig and colleagues as a model of the burning sensation often experienced by patients with neuropathic pain [5], [6], [7].The TGI may for instance be suitable to explore the mechanisms of cold-allodynia, a symptom common in patients with central neuropathic pain of various etiologies. Patients with cold-allodynia report burning sensations when an afflicted area is put in contact with cold objects that otherwise are experienced as simply ‘cold’. Unrelenting spontaneous and/or evoked pain – often having a burning quality - is a common and often treatment-refractory symptom after lesions of the central nervous system [8], [9], [10]. A better understanding of the mechanisms involved in such distressful symptoms may translate into improved treatment. To this end, the TGI has been studied with regard to pharmacologic [11], [12], multi-sensory [13] and affective [14] manipulations and, recently, we reported of a putative genetic component relating to variation in thermal-pain sensitivity [15].

Craig and co-workers have in elaborate neurophysiologic studies in anesthetized cats probed spinal mechanisms relating to the TGI [2]. The work shows that simultaneous application of warm and cold temperatures causes an imbalance between firing of spinal neurons reactive to heat, pinch and cold (HPC) and those only responsive to cold (COLD). In response to the TGI, HPC-activity increased disproportionately compared to COLD. This suggests that the illusion may arise by the supraspinal integration between the two kinds of thermoafferent signals. Based on a conjecture proposed in 1911 by Head and Holmes [16], Craig's “thermosensory disinhibition hypothesis” [17] thus states that HPC-activity is centrally inhibited by COLD-activity and that the TGI leads to a disinhibition (“unmasking”) of HPC-related percepts [5]. Craig et al also conducted the thus far only neuroimaging-study of the phenomenon, reported of in 1996 [7]. Using positron emission tomography (PET) imaging the authors demonstrated activation in the mid/anterior insular cortex in response to thermal stimuli. The authors also suggested a crucial role for the anterior cingulate cortex (ACC) in the TGI. The notion of the ACC-activity as a necessary condition in pain-processing is, however, not settled. Such interpretations are, for example, challenged by later studies suggesting an involvement in response selection rather than actual pain perception [18], [19]. In addition, other studies point to an important role of thalamic hyperactivity in evoked cold-allodynia [20], [21], [22], not observed in this early PET-study of the TGI-phenomenon.

Further investigation of the supraspinal mechanism involved in the illusion is therefore warranted. This is important in evaluating if and how the TGI permits modeling of phenomena involved in pathological pain-states. Methodologically, functional magnetic resonance imaging (fMRI) has certain advantages over PET in studies of functional brain-activity. PET can only yield intermittent measures of activity integrated over some 30–60 seconds, whereas fMRI yields more continuous measures for extended periods of experimentation. This has led to a vastly improved ability to collect statistically valid spatiotemporal data on human brain function. An important step towards attaining an increased mechanistic understanding of the TGI is therefore the development of an fMRI-compatible TGI-unit. This, however, implies certain technical challenges as a stimulus system for use in an fMRI-environment needs to be non-ferromagnetic and not emit any radio-frequent noise that potentially could interfere with the MRI-signals. In addition, it is an experimental prerequisite that the system provides rapid and reliable response-times when shifting between the various stimulus conditions.

The aim of the present study was to develop and evaluate such an MRI-compatible TGI-unit and image the supraspinal correlates of the illusion in a cohort of healthy volunteers. We made several modifications to a TGI-thermode employed in a recently reported study [15] to meet the requirements imposed by fMRI. Twenty-healthy volunteers were recruited. The affective-motivational (i.e. “unpleasantness”) and sensory-discriminatory (i.e. “pain-intensity”) of the TGI and its constituent temperatures were evaluated in the MRI-environment. During acquisition of fMRI-scans cold, warm and TGI- stimuli (each followed by a neutral baseline) were presented.

## Methods

### Participants

The study was approved by the regional ethics committee in Stockholm. Subjects were recruited through advertisement, provided written informed consent and were paid for their participation. Subjects were screened to meet the safety requirements for the MR-environment (e.g. no history of heart or brain surgery, no metal implants/braces, not pregnant). Furthermore, subjects were required to be right-handed and healthy, without any self-reported history of present or past pain- or psychiatric disorder. Participants were recruited to balance sex. Twenty right-handed volunteers (10 males, 10 females) were tested. One additional subject was enrolled in the study but excluded from all analyses because of presenting with a marked pain-response to mild cold stimuli during sensory testing. Apart from contraceptives, intake of any pharmaceuticals - with the potential to influence pain perception - was not allowed within 48 hours of the experiment. To the best of our knowledge, subjects were naïve to the TGI and had not previously participated in pain-experiments conducted by our group. Care was taken when briefing subjects about the experiment. Prospective participants were merely told that the experiment would involve “the application of different temperatures that could be perceived as painful and/or unpleasant but that the stimuli would not be harmful”.

### Thermal sensation and pain thresholds

Subjects were comfortably seated in a 3-sectioned clinical examination bed. A computer controlled Peltier-type thermode system was used (PATHWAY, model ATS, Medoc, Israel). The active surface (30×30 mm) of the thermode was attached to the skin overlying the left calf muscles using a Velcro strap. The subjects were instructed to respond using a button held in their right hand. Baseline was set at 32.0°C. For assessment of thermal detection, a change rate of 0.5°C/s and a return rate of 8.0°C/s were used. The end-to onset inter-stimulus interval was 15 seconds. For thermal pain measurements a change rate of 1.5°C/s and a return rate of 8.0°C/s were used and the end-to onset inter-stimulus interval set to 30 seconds. Firstly, two thresholds for warm sensation were evaluated. After this, heat-pain thresholds were assessed and subjects were instructed to respond to the “slightest percept of pain”. The thermode was then moved to a different skin area and a similar test for cold-sensation and cold-pain was performed. For cold-pain testing the system had a lower limit of 0°C. If 0°C was reached before pain had been perceived (i.e. the button pressed) the program automatically returned the thermode temperature to baseline. If this happened a threshold of 0°C was assigned to the present and any pending trials.

### Ratings of the thermal grill illusion

VAS-ratings of the TGI as well as its constituent cold and warm temperatures were collected immediately prior to fMRI. This was done with the subject in the supine position on the MRI-gurney, with the thermal grill positioned as described below, but outside the MRI-gantry. The order of the stimuli was randomized and counterbalanced. Both the experimenter and the subject were blinded to the order of the three different kinds of stimuli (warm, cold, warm+cold = TGI). Each stimulus lasted for 30 seconds and the subjects were asked to provide ratings 15 seconds into each stimulus. To approximate the conditions during the imaging, the thermoneutral inter-stimulus-interval (ISI) was 20 seconds. For each stimulus, the affective-motivational (i.e. “unpleasantness”) and the discriminative-sensory dimensions (i.e. “pain-intensity”) [23] were rated on two separate 100 mm VAS-scales printed on the same sheet of paper. Subjects were instructed to rate any “unpleasantness irrespective of pain” and the “pain intensity irrespective of unpleasantness”: (‘no pain’ [left]- ‘worst pain imaginable’ [right]) and (‘not unpleasant’ [left]- ‘the most unpleasant feeling imaginable’ [right]).

### The thermal grill

The thermal grill was based on a prototype previously used by our group in a in a behavioral study [15]. Compared to the prototype version, the TGI-unit used in the present experiment was designed specifically to allow rapid switches between the different temperatures and a neutral baseline.

#### Apparatus

The stimulation surface consisted of eight 1.0 mm thick jewelers’ grade silver bars, each measuring 11 mm by 80 mm. The bars were placed side-to-side with a spacing of 2.4 mm, in a Perspex-housing, giving a total stimulation surface of 80 mm by 105 mm.

**Figure 1:**
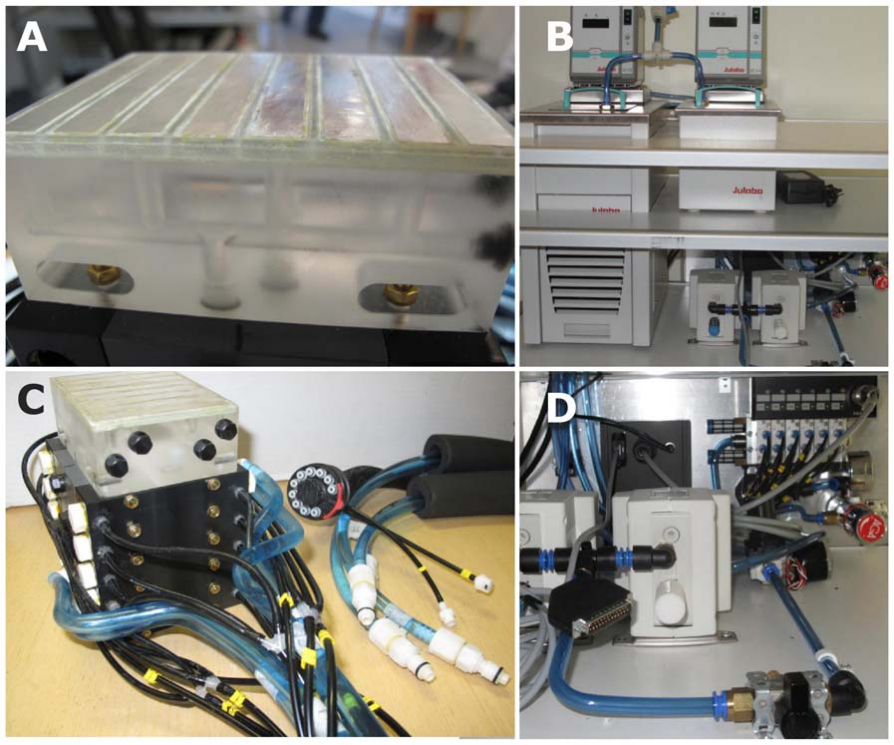
The thermal grill-unit. **Panel A**) Detail of the surface of the thermode, consisting of eight silver bars each measuring 11 mm×80 mm. The bars were mounted onto a Perspex-block bringing odd and even numbered bars into direct contact with (potentially) different pools of circulating water. Channels drilled through the Perspex-block connected to the inlet/outlet unit (grey-colored block) through the bottom of the thermode. **Panel B**) Water baths for cooling and warming, connected to a circulation pumps. **Panel C**) The TGI-thermode attached to the grey Delrin inlet/outlet unit. The Delrin-block has bored channels in which ten pneumatically operated piston-valves operate. The two segregated pools of warm- and cold-water are continuously circulated through the unit (blue hoses). The position of the valves, set through computer controlled pneumatics (thin black tubes), determine the water-flow to the Perspex-block thermode. The thermode and inlet/outlet unit are connected to the baths, pumps and control devices through a thermally insulated umbilical-cord running through the radiofrequency wave-traps of the MRI-Faraday cage. **Panel D**) Pressurized air-operated process pumps for circulating the water, along with computer-controlled pneumatic switches – allowing precise timing of stimulus-presentation.

Our TGI-unit can be conceptually divided into the following components: A) thermal baths with circulation-pumps; B) TGI-thermode; C) inlet/outlet-unit fitted to the thermode-head for temperature selection via pneumatics; D) computer control of the temperatures via pneumatic switches. The thermode and inlet/outlet unit were connected to the baths, pumps and control devices through an umbilical-cord running through the radiofrequency wave-traps of the MRI-Faraday cage. See Figure 1.

- Thermal baths with circulation-pumps: Two state-of-the-art thermostat-controlled baths were used for cooling and heating the water, respectively (models F25-ED and EH-5, JULABO Labortechnik GmbH, Seelbach, Germany). Process pumps (model PA3120-F03, SMC corp., Tokyo, Japan) running on pressurized air were connected to each bath via plastic tubing. The pumps were used to draw water through the thermode-unit; creating a vacuum on the outlet-side, rather than pumping water through the unit. This way water could safely and efficiently circulate through the unit with no risk of a leak inside the MR-gantry in case of e.g. accidental hose detachment. The length of each tube, running from the baths in the MRI-control-room to the thermode-unit, was approximately 8 meters. In order to prevent heat-loss/gain the warm and cold tubing were separately insulated. This further stabilized the temperatures by means of counter-current exchange. See figure 1, panel B and D.
- TGI-thermode: The thermode surface consisted of thin pure-silver plates. Silver was chosen as it is non-ferromagnetic and has excellent thermal conductivity. The silver plates were housed in a polymethyl methacrylate (i.e. “acrylic glass”/“Perspex”) unit. The housing was compartmentalized such that even- and odd-numbered silver plates could be put in direct contact with (potentially) different pools of circulating water as selected via the inlet/outlet unit. See figure 1, panel A
- Inlet/outlet-unit: A water inlet/outlet unit constructed from Delrin and PVC was fitted beneath the thermode- housing. The purpose of this was to allow rapid switching of stimulation-temperatures. Water circulated continuously through the system, providing access to stable temperatures. A valve-system guided water flowing in separate channels within the unit. Ten pneumatically operated piston-valves ran in bored channels. Depending on the configuration of these valves, water circulating through the unit could be selected to either pass through the thermode-housing on its circulatory path, or simply be shunted without entering the thermode-unit. Warm and/or cold temperatures could thus be selected to enter the thermode-housing through two separate inlets (one for odd-numbered silver bars, one for even-numbered) – allowing the application of the three conditions (warm-only, cold-only, warm and cold = TGI). See figure 1, panel C. To achieve a thermally neutral baseline stimulus at the silver-plates the “opposite” water bath was selected for a short and calibrated time (0.8–2 seconds) for odd and even numbered plates respectively. This was followed by a complete shunt in the input/output unit resulting in a neutral temperature (i.e. 31.0°C±2.0°C) equilibrating with skin-temperature as verified by pilot testing. These switch-times were included in the total stimulus-length as reported below
- Computer control: Pneumatic switches (model MEH-5/2-1/8-P-L, Festo, Germany) connected through tubing to the inlet/outlet-unit. The electronic control valve for the switches was connected to the parallel-port of a computer. The computer allowed the appropriate combination of valve-positions to be selected depending on desired stimulus type. See figure 1, panel C and D

#### Temperature calibration and response-times

**Figure 2:**
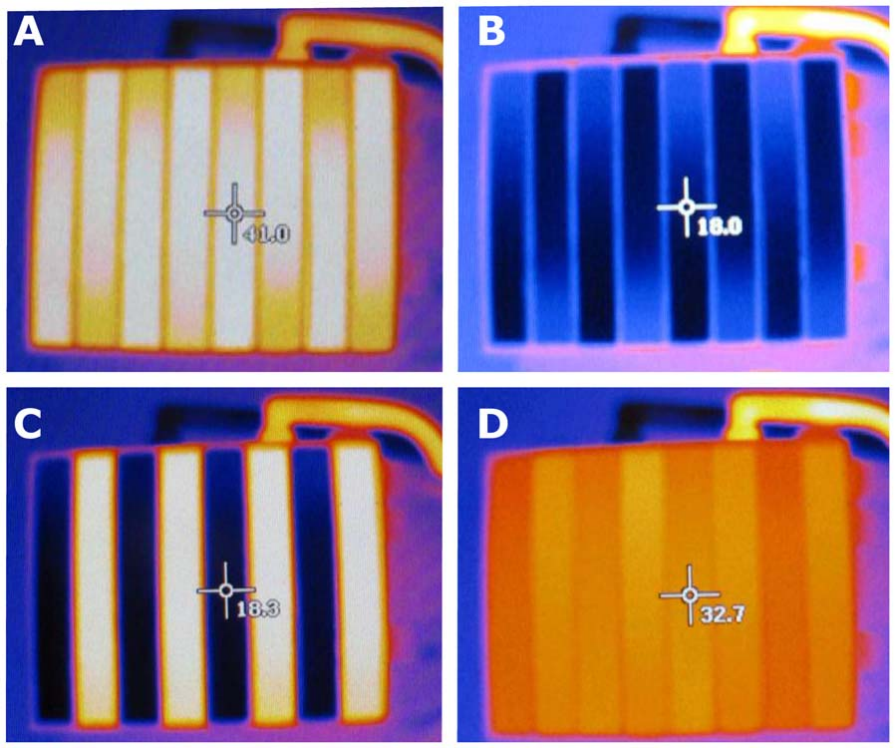
Thermography of the TGI-thermode surface. An infrared (IR) camera with high thermal resolution was used to verify the stimulus temperatures. Note that the temperature shown is that at the crosshair. **Panel A**) Warm-stimulus (41.0°C±0.5°C) **Panel B**) Cold-stimulus (18.0°C±0.5°C) **Panel C**)TGI-stimulus (41.0°C±0.5°C & 18.0°C±0.5°C). Note that the crosshair is centered on a cold plate . **Panel D**) Neutral baseline stimulus (31.0°C±2.0°C).

The water-baths provide an excellent thermal stability of the water supplied to the thermode: within ±0.03°C as specified by the manufacturer. During scans, the temperature displays of these water-baths were monitored from the MRI control-room. The baths were calibrated such that the silver-plates reached the desired temperatures. An infrared (IR) camera with high thermal resolution (model 882, Testo AG, Lenzkirch, Germany) was used. To achieve an emissivity coefficient close to 1.0, thin opaque tape was attached to the silver surface of the TGI-thermode. We thereby minimized the reflection from adjacent IR-sources. The thermode-unit was thus calibrated to 41.0°C±0.5°C, 18.0°C±0.5°C and 31.0±2.0°C. See Figure 2. The temperature levels were verified during several pre-experimental tests with the IR-camera during both single-temperature stimuli as well as TGI stimulus. The system was monitored for the duration of several consecutive experimental sessions, without deviation.

**Figure 3:**
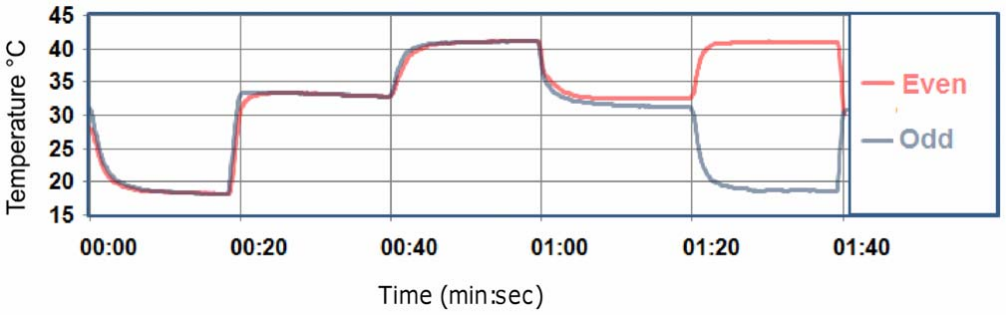
Temperature profiles of juxtapositioned silver bars during stimulation. Representative recordings from two adjacent silver bars (i.e. odd- and even- numbered) of the TGI-system. The recordings were made simultaneously using two infra-red thermosensors with a high temporal resolution. Each stimulus lasted 20-seconds. The graphs show an initial cold-stimulation (18.0±0.5°C), followed by a return to neutral baseline (31.0±2.0°C). This was followed by warm (41.0±0.5°C) and then baseline. Finally, the graphs show the TGI-condition (lasting from 01:20 to 01:40 mins) where adjacent bars have cold and warm temperatures, respectively.

Rise and fall times were determined with an IR-probe with a 9 ms response time (model LT15F, Optris GmbH, Berlin, Germany). The thermal time constant (i.e. time to reach approximately 63% of its desired temperature) was thus determined to be 1.0 second for the TGI-condition, and 1.5 seconds for both warm- and cold-only conditions. The reason for the slightly lower time constant for the TGI is that during this condition water from each bath is only circulated through half (i.e. only to odd or even numbered elements) of the thermode-unit. See Figure 3.

After this initial calibration, the proper functioning of the unit was controlled prior to each experimental session using a calibrated and highly sensitive surface probe with a spring-loaded thermocouple strip (model 925 with probe type-K, Testo AG, Lenzkirch, Germany - calibrated by Nordtec Instrument AB, Gotheburg, Sweden).

#### Thermography: Supplementary online material

As an illustration of the dynamics of the TGI-system, a representative recording of a thermography session is provided. See “Supporting Information Files” – Video S1.

#### Choice of stimulation site

Considering the MRI's relatively narrow gantry the leg was an ergonomically preferable stimulus-site as compared to e.g. the ventral forearm. The skin overlying the left calf-muscle was therefore chosen as stimulus site. It should be noted that the thermal sensitivity of this area is likely to be slightly reduced compared to that of the forearm or hand [24].

#### Choice of cold and warm temperatures

Craig and co-workers PET-imaging study used grill elements set at 20°C and 40°C to induce the illusion [7]. A study by Bouhassira and colleagues suggested that the strength of the TGI-sensation is related to the magnitude of the differential between cold and warm temperatures [25]. We expected the thermal sensitivity to be slightly reduced over the calf as compared to the hand or ventral forearm commonly used in behavioral testing of the TGI [24]. We therefore slightly increased this gap (i.e. 41°C−18°C = 23°C) as compared to the 20°C gap used by Craig and co-workers. Importantly, as revealed by the testing of thermal-pain thresholds, our TGI-temperatures remained well-within the span of innocuous temperatures.

### fMRI-experiment

The experimental scans were carried out in a standard hospital MRI-environment at the Karolinska University Hospital, MR-Centrum in Solna, Sweden. The ambient temperature in the MRI-room during the experiments averaged 21.6°C (range 20.2–22.0°C) with a relative humidity between 40 and 55 percent. As mentioned, the psychophysical assessment of the TGI occurred in the MRI-room immediately prior to scanning, mitigating the potential for environmental confounds in this regard.

Subject rested their left leg on the surface of the thermal grill such that the skin overlying the calf-muscles was in contact with its stimulating surface. The long axes of the silver bars were placed orthogonally to the leg. Cushions were used to ensure that the leg could be comfortably maintained in this position for the duration of the experiment. Images were acquired on a 3.0 Tesla scanner (Discovery MR750, GE) with a 32 channel head-coil (MR instruments Inc). The head was fixated inside the coil with headphones and foam wedges. A 3-plane localizer was followed by an ASSET-calibration for parallel imaging. This was followed by two T_2_*-weighted echo-planar imaging (EPI) scans for fMRI– during which thermal stimuli were applied – each scan lasting 10:20, including 20 seconds of discarded dummy-scans. Imaging parameters were: sequential axial-plane slices with thickness 3.0 mm and spacing of 0.5 mm, 37 slices, 288 mm×288 mm field-of-view (FOV) yielding a voxel-size of 2.25×2.25×3.5 mm^3^, repetition time (TR) 2500 ms, flip-angle 90°, and an echo-time (TE) of 30 ms. The fMRI-scanning was divided into two sessions to ensure optimal subject cooperation. After a brief verbal confirmation that the subject was comfortable, the second session commenced. For each EPI-scan one of five possible stimulus-files was chosen in a randomized and counterbalanced way. Each file contained a different pseudorandomized stimulus order ensuring that not more that two of the same type of stimuli could occur consecutively. Following the 20 seconds of dummy-scans, the first stimulus was applied. Each stimulus lasted 20 seconds and was followed by a 20 second neutral baseline. During each scan, every stimulus type (i.e. cold, warm TGI) was delivered five times each.

It should be noted that we chose to have a neutral baseline condition between each active stimulus. Although this leads to a relative over-sampling of the baseline as compared to each of the three stimulus conditions we found it necessary in order to minimize the risk of peripheral and/or central sensitization as well as to avoid evoking sensations of “paradoxical heat” [3] – by going from warm to cold directly - not caused by the TGI itself.

### Statistical analyses

#### Analyses of behavioral data

SPSS Statistics 17.0 (SPSS Inc, Chicago, USA) was used for analyses. Two-tailed tests were used unless otherwise stated and p-values <0.05 were considered significant. Data are reported as means ±1 standard deviation (SD) and graphs are shown as means with error bars ±1 standard error of the mean (SEM). Shapiro-Wilk tests were used to assess significant deviations from the normal distribution and non-parametric tests (exact) were used when suitable. For the TGI, VAS-ratings of the affective (i.e. “unpleasantness”) and sensory (i.e. “pain-intensity”) components were analyzed separately. Friedman's non-parametric ANOVA was used to compare the ratings of the respective dimension for the TGI with the ratings for cold and warm. These ANOVAs were followed by post-hoc testing using Wilcoxon's signed rank test. Spearman's correlation coefficient was used for the exploratory correlations between activation intensity and VAS-ratings.

#### Analyses of fMRI-data

SPM8 (Wellcome Trust Centre for Neuroimaging, University College London, UK) was used to analyze the fMRI-data. Realigned EPI-images were normalized to the canonical EPI-template in the standard Montreal Neurological Institute (MNI)-space. Normalized EPI-images were smoothed using an 8 mm full-width-at-half-maximum (FWHM)-kernel. For each individual subject, first-level analyses were carried out using a fixed-effects analysis (FFX), i.e. compounding both scans into the same general linear model (GLM). Using a boxcar-model, onsets and durations for the application of cold, warm, TGI and neutral temperature stimuli were entered as regressors. Movement parameters provided by the realignment process were added as covariates of no interest. These explanatory variables were convolved with the canonical hemodynamic response function (HRF) in the GLM design matrix. The following T-contrasts were estimated: all stimuli (cold, warm or TGI) versus neutral baseline, cold versus neutral, warm versus neutral, TGI versus neutral, and TGI versus areas activated by both cold and warm. Group-level analyses were conducted using the summary statistics approach, to achieve a random-effects analysis (RFX), using one-way t-tests. The resulting maps were thresholded at a voxel-level of p<0.001.To achieve an overall α-level<0.05, corrected for multiple-comparisons, we used the program REST v 1.0's (http://www.restfmri.net) instantiation of AlphaSim (http://afni.nimh.nih.gov/pub/dist/doc/manual/AlphaSim.pdf) applied to the 123507 voxels identified by a whole-brain mask. One-thousand Monte-Carlo simulations were conducted and the extent of the needed cluster-size was determined to be ≥40. All contrasts were evaluated at this corrected α-level<0.05 (i.e. p<0.001, cluster-size ≥40). Additionally, we conducted an exploration/sensitivity-analysis of these contrasts at the less stringent cluster-size of ≥20. Results are displayed on the standard MNI152 average T1-weighted brain. We employed the Anatomy Toolbox v 1.8 [26] to assist in the localization of significant clusters. The MNI-coordinates (x, y, z in millimeters) of the peak-level voxels of these clusters are reported. For exploratory correlation analyses between brain activity and subjective ratings, we used the MarsBaR region of interest toolbox for SPM (version 0.42) to extract the weighted beta-values averaged across voxels in regions-of-interest (ROIs).

## Results

### Thresholds of thermal sensation and pain over the left calf

Subjects detected warm at an average of 35.5°C±2.1 and cold at 27.6±1.7°C respectively. The average heat-pain threshold was 45.0°C±1.8. Fourteen of the twenty subjects did not report cold-pain against the skin overlying the calf for the test reaching 0°C; the average cold-pain was 3.8°C±7.5°C. This served as a confirmation that our TGI-temperatures were well within the range of innocuous temperatures for the subjects.

### Behavioral validation of the TGI

**Figure 4:**
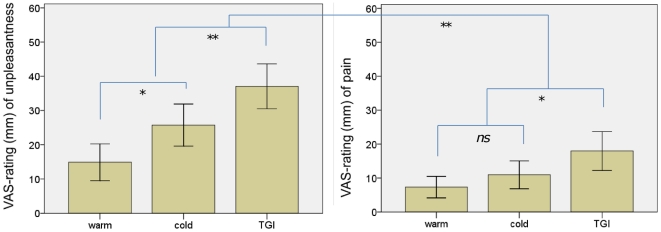
Ratings of the TGI, collected prior to fMRI. Affective-motivational (“unpleasantness”) and sensory-discriminatory (“pain-intensity”) dimensions were rated on separate 100 mm-long VAS scales. Significance levels: ** = p<0.01,* = p<0.05, ns = non-significant.

With regard to ratings of unpleasantness the cold, warm and TGI differed significantly [χ^2^(2) = 17.1, p<0.001]. Post-hoc comparisons revealed TGI ratings as significantly more unpleasant than both cold [z = −2.5, p = 0.01] and warm [z = −3.6, p<0.001]. Additionally, cold was rated as more unpleasant than warm [z = −2.3, p = 0.02]. With regard to pain-ratings, the three conditions differed only by a non-significant trend [χ^2^(2) = 5.8, p = 0.051]. Given the strong a-priori expectations we nonetheless conducted post-hoc tests. These revealed that the TGI was rated as more painful than both cold [z = −2.0, p = 0.04] and warm [z = −2.5, p = 0.01], with no difference in ratings between warm and cold [z = −1.0, p = 0.35]. As also expected from previous results [15], the TGI was rated as more unpleasant than painful [z = −2.9, p = 0.002]. See Figure 4.

### fMRI-analyses

**Figure 5:**
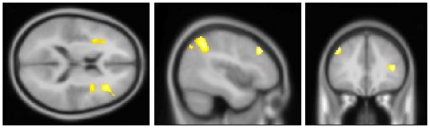
Overall contrast of stimulation versus neutral baseline. One-third of trials were cold, 1/3 warm, and 1/3 TGI. The anterior insula was activated bilaterally along with bilateral fronto-parietal areas. Crosshair position: x = −43 mm, y = 33 mm, z = 16 mm. See also Table 1.

**Figure 6:**
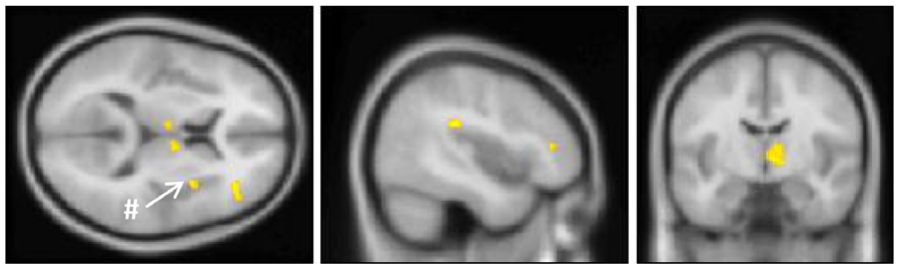
TGI-stimulation versus neutral baseline. Crosshair-position: x = 44 mm, y = −8 mm, z = 10 mm. A large cluster of activation was observed in the right thalamus, partially extending over into the left thalamic region. Right-sided fronto-parietal activation was also found. #: the post-hoc contrast with less stringent cluster-extent criteria (cluster-extent≥20 voxels instead of 40) revealed an additional activation in the right mid/anterior insula. See also Table 2.

**Figure 7:**
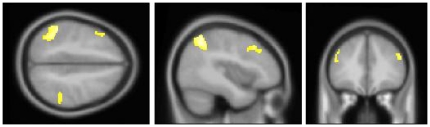
Cold-stimulation versus neutral baseline. Crosshair-position: x = −40 mm, y = 34 mm, z = 45 mm. Significant fronto-parietal activity was observed in response to cold-stimulation but unlike the TGI, no thalamic-activity emerged. See also Table 3.

**Figure 8:**
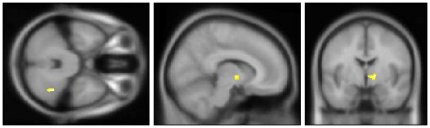
TGI versus cold and warm. Crosshair position: x = 10 mm, y = −3 mm, z = −32 mm. This corroborated the TGI-related activation in the right thalamus. Additionally, activation was observed in the right hippocampal formation (not shown) as well as in the right cerebellum. See also Table 4.

To validate the functioning of the stimulus-device and timing on the experimental paradigm as a whole, we first calculated the contrast between the application of any thermal stimulus (cold, warm or TGI) and the neutral baseline. The contrast revealed bilateral activation in the anterior insulae together with fronto-parietal activity. See Table 1 and Figure 5. We then evaluated the specific contrasts between the TGI, cold, and warm stimuli –respectively – versus the neutral baseline. For the TGI, a large cluster of activation was observed in the right thalamus, partially extending over into the left thalamic region. Additionally, right-sided fronto-parietal activation was found. For the exploratory contrast at a less stringent cluster-extent criteria (i.e. p<0.001, with a cluster-extent≥20 voxels instead of 40), activation in the right mid/anterior insula emerged. See Table 2 and Figure 6. For the cold, no thalamic activity emerged. Instead, this contrast revealed bilateral activation of the inferior and middle frontal gyri and bilateral activity in the inferior parietal lobuli. See Table 3 and Figure 7. No clusters of activations survived thresholding in the warm versus neutral contrast. Finally, we evaluated the contrast of the TGI versus areas commonly activated by warm and cold. This corroborated the TGI-related activation in the right thalamus. Additionally, activation was observed in the right hippocampal formation as well as in the right cerebellum (corresponding to lobue VIIa). See Table 4 and Figure 8.

**Table 1:** Thermal stimulation versus baseline.

Region	Side	T	Cluster-size	peak-voxel x, y, z (MNI)
IPL (including SMG)	left	5.72	1122	−48, −58, 52
SMG	right	5.06	130	50, −30, 24
MFG	left	6.09	126	−42, 30 , 38
IFG, MFG	right	6.16	316	40, 34, 12
Anterior insula	left	5.31	144	−32, 0, 22
	right	4.47	65	36, 6, 18

*The contrast revealed bilateral activation in the anterior insulae. Additionally, fronto-parietal activation was present in areas corresponding to the right inferior frontal gyrus (IFG) and right supramarginal gyrus (SMG) of the IPL as well as the left middle frontal gyrus (MFG) and left inferior parietal lobule (IPL). See* *Figure 5* *.*

**Table 2:** TGI versus neutral baseline.

Region	Side	T	Cluster-size	peak-voxel x, y, z (MNI)
Thalamus	Right	4.55	270	10, −6, 0
	Left	3.86	(part of above)	−6, −12, 10
SMG	Right	5.65	231	50, −30, 24
IFG	Right	4.39	42	38, 36, 10
Mid/ant insula #	Right	4.31	25	36, 6, 12

*A large cluster of activated voxels was found in the right thalamus along with a portion extending into the left thalamic region. Activation was also seen in the right inferior frontal gyrus (IFG), right superior marginal gyrus (SMG). For the exploratory contrast at less stringent cluster-extent criteria, activation in the mid/anterior insula was also observed (#). See* *Figure 6* *and* *9* *.*

**Table 3:** Cold-stimulation versus netural baseline.

Region	Side	T	Cluster-size	peak-voxel x, y, z (MNI)
IPL	left	5.40	816	−38, −60, 54
	right	4.71	163	56, −42, 50
MFG, IFG	left	4.81	294	−48, 28, 34
	right	4.99	58	50, 36, 26

*No thalamic activity emerged. Instead, this contrast revealed bilateral activation of the inferior and middle frontal gyri (IFG, MFG) and bilateral activity in the inferior parietal lobuli. See* *Figure 7* *.*

**Table 4:** Contrast of TGI versus warm and cold.

Region	Side	T	Cluster-size	peak-voxel x, y, z (MNI)
Thalamus	right	4.94	63	12 , −4, −2
Cerebellum	right	5.59	73	40, −58, −38
Hippocampus	right	4.40	224	28, −28, −6

*Along with right-sided thalamic activity, significant clusters emerged in the right cerebellum (corresponding to lobule VIIa) and in the right hippocampal formation. See* *Figure 8* *.*

### Correlations between subjective ratings of the TGI and brain-activity

**Figure 9:**
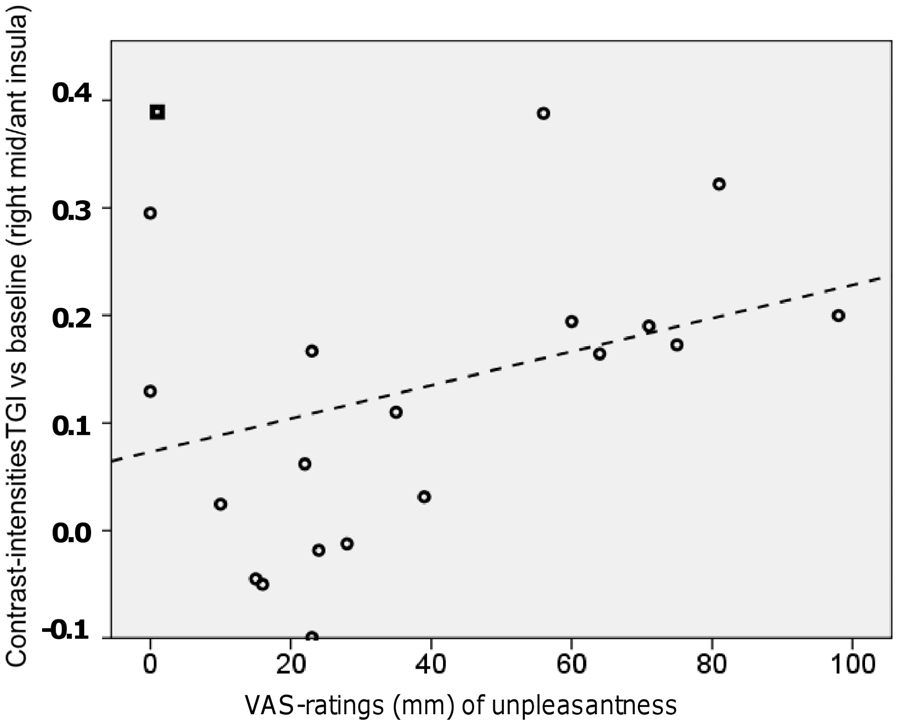
Correlation between ratings of TGI-unpleasantness and right insular activity. The contrast comparing TGI-stimulation against the neutral baseline revealed right mid/insular activity in a 25-voxel cluster. See # in figure 6. This cluster was used to define a functional region-of-interest (ROI) from which the average cluster-intensities (i.e. average contrast- weighted beta-values) were extracted for each individual subject. The figure shows these intensities plotted against each individual's VAS-ratings of “unpleasantness” for TGI, [rho = 0.31, p = 0.09, one-tailed test]; removal of a putative outlier (shown as a square) strengthened this correlation considerably [rho = 0.51, p = 0.01, one-tailed test].

Exploratory post-hoc contrasts revealed an expected [3], [7], [27], [28] activation of the right mid/anterior insular region in response to the thermal grill versus neutral baseline. There is good evidence that this cortico-limbic structure is important for subjectively experienced feeling-states in general [29], [30], and – importantly – the neural processes underlying the feeling of “unpleasantness” [31], [32], [33], [34]. We therefore used a functional region-of-interest approach to explore the relationship between rated unpleasantness and right mid/anterior insular activity. Importantly, the VAS-ratings were made before the acquisition of functional images and did not enter into the GLM-analyses of the fMRI-data, thereby eliminating the possibility of a confounding by the rating-procedure per-se on these correlations. The region-of interest (ROI) was defined by the 25-voxel activated cluster revealed by the group-level contrast. We extracted the contrast weighted intensities (i.e. beta-values), averaged across the voxels in the ROI, for each of the twenty subjects. These intensities were plotted against the individual VAS-ratings of “unpleasantness” for TGI. There was a positive, trend-level significant, correlation between these values [rho = 0.31, p = 0.09, one-tailed test] and removal of one putative outlier strengthened this correlation considerably [rho = 0.51, p = 0.01, one-tailed test]. The outlier was identified post-hoc as the sole observation with a standardized residual error >2. See Figure 9. VAS-ratings of pain did not exhibit such an association with insular-activity [rho = −0.11, p = 0.66]. Similar intensities extracted from the thalamic region did not correlate with the extracted insular intensities [rho = 0.07, p = 0.80] or with ratings of unpleasantness [rho = −0.16, p = 0.50].

## Discussion

### Summary of findings

To evaluate the supraspinal correlates of the thermal grill illusion (TGI) we developed an MRI-compatible thermode capable of rapidly presenting warm, cold and TGI (i.e. juxtapositioned warm and cold) stimuli. The described TGI-unit permits an inherently safe, thermally and temporally reliable presentation of the TGI-stimulus in an MRI-environment. With regard to ratings of the affective-motivational (“unpleasantness”) and sensory-discriminative (“pain”) dimensions of the TGI, we largely replicated our previous behavioral results using a related thermode-prototype [15]. The TGI was rated as significantly both more unpleasant and painful than each of its constituent cold (18°C) and warm (41°C) temperatures by themselves. Additionally, the TGI was rated as more unpleasant than painful. The most salient feature revealed by the fMRI-analyses was the specific and strong activation of the contralateral thalamus by the TGI as compared to the patterns of activations provided by its constituent temperatures. As thalamic aberrations appear to play a key role in central pain syndromes [16], [20] this finding may suggest an important overlapping mechanism of the TGI and such pain-pathologies.

As an evaluation of the overall stimulus-paradigm, a contrast of all thermal stimuli (compounding TGI, cold and warm) versus the neutral baseline revealed activation in bilateral frontal gyri as well as the inferior parietal lobuli (on the right side limited to the supramarginal gyrus) together with a bilateral activation of the anterior insulae. Extensive evidence exists that the neural representation of homeostatically relevant feeling-states are encoded in the insulae [27], [29], [35] and both noxious and innocuous warm and cold stimuli have been reported to activate a network involving this region [36]. The fronto-parietal activation observed for the overall, cold and TGI-contrasts may well be related to attention-orienting effects [37], [38] in response to salient environmental stimuli. It is also worth noting that the parietal activation in response to the TGI-stimulus was located more ventrally, i.e. in the supramarginal gyrus (SMG), as compared to the cold-stimulus. The TGI-activity was also lateralized to the right. Accordingly, the right SMG has been suggested to be part of a network (also including the right inferior frontal gyrus, right anterior insula and anterior cingulate cortex) activated by unexpected/novel stimuli [38].

The sensitivity in the comparison of each of the types of stimuli with the neutral baseline was reduced by the limited number of epochs (10 per type per subject). Despite this, the TGI evaluated against the neutral baseline displayed a strong activation in the contralateral thalamus, not seen in the overall contrast. At the slightly more liberal cluster-threshold, the TGI-contrast also revealed activation in the right mid/anterior insula. In contrasting cold with neutral, only the fronto-parietal activation was seen and no clusters of activation survived thresholding for the contrast of warm versus neutral. Finally, contrasting the TGI versus areas commonly activated by warm or cold corroborated the activation in the right thalamus.

### Interpretations of TGI-induced thalamic activity and an alternative TGI-hypothesis

The TGI-related thalamic activity was most expressed contralateral to the stimulation. As far as we know, this is the first neurophysiological evidence for thalamic involvement in the TGI. Craig and co-workers proposed the TGI as an experimental model of central neuropathic pain [2], [6], [7], providing a major impetus for the presently reported study. It is therefore noteworthy that a key finding in imaging studies of central neuropathic pain involves thalamic aberrations. An important caveat in this regard is that an overlapping activation pattern between the TGI and neuropathic pain must not be taken as evidence that the two are equivalent. Bearing this in mind, a comparison between the TGIs supraspinal correlates and those found in studies of e.g. evoked allodynia could nonetheless help to generate clinically relevant hypotheses.

Whereas the thalamus may be hypoactive during rest in patients with central pain, hyperexcitability has been found during evoked allodynic pain – possibly relating to a loss of inhibitory thalamic neurons – as reviewed by Veldhuijzen et al [20]. A pioneering study by Cesaro et al using single-photon emission computerized tomography (SPECT), demonstrated a thalamic hyperactivity in response to allodynia following central post-stroke pain (CPSP) [22]. Also, a similar neural signature to that presently evoked by the TGI was found in a PET-study by Peyron and colleagues. The authors studied evoked cold-allodynia in nine patients with infarction of the lateral medulla resulting in Wallenberg's syndrome [21]. The allodynic response was coupled to increases in cerebral blood-flow in the contralateral thalamus, post-central gyrus and inferior parietal lobule as well as anterior insular and medial prefrontal cortices. As the allodynic symptoms were unilateral, it was possible to study the non-allodynic cold-response in the same set of participants. Whereas this stimulation increased the signal in the ipsilateral inferior parietal lobule and inferior frontal gyrus, it did not significantly modify blood-flow in the thalamus.

Thermosensory information from the superficial lamina of the spinal dorsal horn is relayed in the spino-thalamic tract (STT) to the thalamus. A thalamic involvement in the TGI is compatible with the overall concepts underlying Craig's proposed thermosensory disinhibition hypothesis, postulating a TGI-induced central unmasking of burning pain normally inhibited by cold [5]. Regarding the thalamic nuclei involved, Craig posits a unique role for the so-called posterior part of the ventral medial nucleus (VMPo) of the thalamus in subserving an afferent homeostatic pathway shared by pain and temperature [39] and – consequently – the TGI [7]. This matter is controversial [40], [41], and Graziano and Jones have presented evidence “disproving the existence of the VMPo as an independent thalamic pain nucleus” [42]. As reviewed by Ralston there is indeed strong evidence that the largest somatosensory nucleus – the ventrocaudal nucleus (Vc) – receives lamina I afferents carrying nociceptive and thermal information [41]. Accordingly, the Vc is implicated in central pain following thalamic stroke [43]. The resolution of the presently reported results does not permit an exact localization of the observed activation to specific nuclei.

The demonstrated activation of the thalamus by the TGI suggests an increased computational load, rather than a simple “TGI-related-relay”. One putative explanation of the thalamic response to the TGI is therefore that it reflects alterations in certain thalamo-cortico-thalamic loops. Pathological reverberations in these loops have been suggested by Llinas [44] to underlie central pain phenomena. A key mechanism in the appearance of these “dysrythmic” loops is believed to be aberrant so-called low-threshold calcium spike (LTS) bursts by thalamic neurons [44]. LTS-bursts are caused by a de-inactivation of thalamic calcium channels by membrane hyperpolarization (i.e. inhibitory events) and underlie normal thalamic oscillations [45]. The same thalamic neuron can switch between by such LTS-bursting activity (at low membrane potentials) to graded repetitive firing at higher potentials [45], [46]. Altered LTS-bursting has been coupled to a variety of neurological disorders, including neuropathic pain [47]. Importantly, thalamic projection neurons both send and receive inhibitory feedback to control their firing in relation to afferent volleys; excitatory signals to thalamus therefore also lead to inhibitory activity. Accordingly, sensory stimulation is capable of inducing LTS-bursts, through inhibitory membrane-hyperpolarization [46], [48]. As elegantly demonstrated by Lee and colleagues, human thalamic Vc-neurons responsive to cold have been found to have particularly high rates of such LTS-mediated firing in response to stimulation, irrespective of stimulus-type [49].

Hypothetically, the warm channel of the TGI may interfere with these normal processes. We therefore suggest that TGI-phenomenon could arise from warm-related *additional inhibition* (over and above that induced by inhibitory-feedback of the cold-stimulus alone) of such thalamic cold-responsive neurons and thereby an altered (i.e. increased) LTS-burst activity compared to that caused by application of cold alone. Such putative warm-induced membrane-hyperpolarization may for example be present in the form of lateral-inhibition [50], [51], [52], [53], under normal circumstances having a functional thermo-discriminatory role with regard to the graded repetitive firing at more depolarized membrane-potentials. This “over-inhibition” hypothesis is testable: microelectrode recordings from awake patients receiving thalamic implants [49], or unanesthetized monkeys [54], [55], could be obtained during TGI-stimulation.

Such an “over-inhibition” hypothesis appears compatible with the general idea of thermosensory convergence/addition advanced by Bouhassira and colleagues based on the finding that a lowering of the cold temperature used to elicit the TGI has comparable perceptual effects to an increase in warm temperature [56]. Perhaps owing to the paucity of literature on lateral-inhibition and thermal sensation [51], [52], the concept has – to the authors' knowledge- so-far only been mentioned very briefly in relation to the TGI-phenomenon [57].

### Insular and cerebellar activation

The anterior part of the insula is a region of particular interest with regard to subjective feeling-states [27], [29], [35] and interoceptive awareness [28], [58]. Comparing the TGI against baseline revealed activity in the right mid/anterior insula. Consistent with the region's proposed role we demonstrate a positive correlation between the individual activation-intensities and subjective ratings of “unpleasantness”. For the present purposes it is also interesting to note that the perception of a phenomenon related to the TGI – namely “paradoxical heat” [4] – has been specifically tied to activity in the right anterior insula in a percept-related fMRI-study [3]. When contrasting the TGI against activation common to both cold and warm the insular activation did not survive (exploratory) thresholding, probably due to a partially overlapping activation, albeit stronger in the TGI. Instead, however, a cerebellar activation contralateral to the stimulus emerged. Such cerebellar activity is often seen in studies involving actual noxious stimuli [59]. One interpretation is that it may relate to withdrawal behavior/reflexes [60]. As the activation did not emerge in relation to the neutral baseline, however, this is not a likely interpretation here, however. Instead, the cerebellar activation may reflect aversion-related processes that differed in sign between the TGI and control temperatures. Accordingly, the work by Moulton and colleagues suggests that the cerebellum contains regions related to the processing of aversive emotional information [61].

### Lack of TGI-related ACC-activity, methodological considerations

Unlike the PET-study of the TGI by Craig and co-workers [7], we did not observe any activation in the anterior cingulate cortex (ACC). PET-imaging revealed an ACC-response both to the TGI and noxious thermal stimuli, whereas no such response was present for the innocuous warm and cold temperatures alone. Accordingly, the authors contend that ACC activation is “an integral component of the neurobiological basis of the thermal grill illusion of pain” and that this activation supports the TGI's use in pain-research [7]. Although implicated in processing of pain-unpleasantness [62], the specific interpretation of anterior cingulate activation is, however, not settled. ACC-activity is not a consistent finding in studies of pain in general [63], [64], or in evoked allodynia in particular [20], [63]. For example, whereas a study of evoked cold-allodynia in syringomyelia patients did indeed display ACC-activation, the same patients did not exhibit this when tested for tactile allodynia [65]. It has been proposed that activity in the ACC seen in pain-paradigms may reflect response behavior and generation of (affective) decisions rather than sensory processing [18], [19].

The inconsistencies may in part be related to different paradigms and instructions to participants. Simple rating-tasks performed during neuroimaging of salient emotional stimuli have been demonstrated to influence brain-activity (e.g. ACC-activity) [19]. In Craig's study participants rated the stimuli during acquisition of PET-images [7]. In our study, the rating was conducted just prior to scanning. Our subjects were not instructed as to whether or not to attend to the stimuli - apart from being told that it was important that they remained still and awake. Specifically, subjects did not know that the TGI-sensation was the stimulus of primary interest – possibly reducing “response-selection” related activity. Furthermore, differences in data-processing may contribute to discordant findings. As was common in the mid 1990's, Craig and co-workers study used an average map of all activation – corresponding to a fixed-effects model (FFX). Importantly, this implies that regional activity specific in a subset of participants may drive the main effect provided it is large enough. In the present study, however, we used the now available and more appropriate random-effects model (RFX) permitting more rigorous inference back to the population of interest (i.e. healthy right handed volunteers) as for the studied BOLD-activation (see e.g. http://www.fil.ion.ucl.ac.uk/spm/doc/books/hbf2/pdfs/Ch12.pdf). Our results should not be taken as an indication that the TGI does not significantly activate the ACC above baseline, but rather that it does not do so consistently enough to be generalized to a healthy population given the exact instructions and paradigm used in the present study.

### Affective-motivational and sensory-discriminatory dimensions of the TGI

The temporal dynamics of the TGI are also likely to influence the results. In fact, these temporal dynamics may relate to the whether or not the grill is an illusion of “pain” as such. Leung and co-workers demonstrate a time-course variation of the TGI, such that it was rated as more painful than its constituent temperatures at 3 seconds into the stimulus but not at 10 seconds [66]. Such findings may underlie some of the discordant findings; for example, Frusthorfer et al conclude that the TGI produces “synthetic heat” but that it is not “painful” [4]. Craig et al let warming precede cooling in the TGI stimulus by 5 seconds to increase the effect (footnote 7 in [5]) and it has been shown that such preheating facilitates the perception of “synthetic heat ” due to cooling of the skin [67], possibly increasing the pain-intensity as well. Our present, and previous [15], results do however support the notion put forth by Craig et al [5], and later corroborated by other groups [25], [68], [69], that the TGI may indeed be classified as “painful.” Additionally, our data indicate that perceptual quality of the TGI lies more along the affective-motivational than sensory-discriminatory dimension. Accordingly, whereas ratings of “unpleasantness” correlated with activation-intensities in the right mid/anterior insula – no such association emerged for ratings of “pain-intensity”. These correlations cast additional light on the interpretation of some of our previously reported findings: we recently reported of a putative dissociation between TGI-unpleasantness and TGI-pain on the basis genetically inferred differences in the serotonin-system and gender [15]. Importantly, being more an “illusion of unpleasantness” or dysesthesia than of “pain” does not disqualify the TGI as an important tool in probing the mechanisms of, for example, cold-allodynia and/or dystesthesia related to central lesions. Such percepts may be functionally limiting, regardless of whether or not they are described as painful [70]. In patients with Wallenberg's syndrome, for instance, the application of cold to an afflicted area has been described as a “new, strange and extremely unpleasant feeling” [21].

### Study limitations and future perspectives

As with all imaging studies, it is imperative to be cautious of the interpretation of “reverse inferences” [71]. For instance, an overlapping activation pattern between the TGI and that seen in evoked cold-allodynia must not be taken as hard evidence that the two are equivalent. A more prudent use of such common activation patterns is to generate mechanistic hypotheses as well as to complement studies using other methodologies. As with all fMRI-studies, the BOLD-signal is a surrogate signal related to blood-flow increases induced by mass-activity in large populations of neurons and inferences as to the specific neural architectures involved and relationship between inhibitory and excitatory mechanism cannot be made [72]. It would therefore be highly relevant to collect thalamic microelectrode recordings to assess the influence of the TGI-stimulus on LTS-burst activity. That is, to test our ‘thalamic over-inhibition hypothesis’ as outlined above.

It is also important to note that neuropathic pain constitutes a heterogenous group of disorders and that mechanisms underlying experimentally evoked percepts in healthy volunteers are not necessarily transferable to those underlying evoked allodynia in pain-patients. One obvious caveat in this regard is changes over time: baseline neural activity and chronicity are likely to be related. For example, in as study by Ushida et al, patients with neuropathic pain-onset within 12 months had a hyperperfusion of the contralateral thalamus. This was not observed in patients with pain of longer duration [73]. It is beyond the scope of this paper to discuss the various symptoms and sensory aberrations of different neuropathic pain-states. As reviewed by Moisset and Bouhassira, the existence of a unique “allodynia network” is highly unlikely given the heterogeneity of the conditions [74] – see also [75]. Future studies will hopefully delineate for which conditions the TGI may be an appropriate perceptual/mechanistic-model. For instance, it would be interesting to directly compare the supraspinal correlates of a “real” cold-allodynic response to that of the TGI, presenting the latter in a thermosensory intact area. As there is evidence that lesions of the non-dominant (right) thalamus is associated with a greater risk of pain, a study of laterality-effects of the TGI would also be of value [10]. Additionally, studies of how cognitive factors may modulate the TGI could shed light on the involved perceptual mechanisms. For instance, it is known that expectations modulate pain perception – including allodynia [76]– and the prefrontal cortices (PFC) are important in this regard [76], [77], [78], [79] . Future studies could be designed to assess the interplay between relevant PFC-engaging paradigms, simultaneously gauging the subjective ratings of the TGI-percept together with the thalamic activity. Studies aimed at assessing the functional connectivity between supraspinal areas are also feasible.

While our data delineate supraspinal mechanisms involved in the phenomenon, the present study should not be interpreted as evidence that the TGI only has supraspinal correlates. There is evidence that peripheral as well as spinal processes also contribute to the TGI. For instance, Campero and co-workers have reported of human cutaneous C-fibers that are activated by both heating and cooling. As the authors note, the TGI could very well be related to such bimodal receptive properties of the peripheral somatosensory system [80]. Spinally, thermoafferent processing, especially that of the superficial lamina in the dorsal horn [5], [39], is likely to contribute to the TGI and individual variation in how it is perceived. Given the presently reported findings, such spinal processes appear especially relevant to evaluate in humans. Future studies could make use of the presently described TGI-system in a spinal-fMRI setting to explore these mechanisms non-invasively.

A few technical notes should also be made. We used a fixed temperature paradigm and the perceived intensity of the TGI is known to vary quite substantially [15], [56], [81]. Further studies, especially those evaluating the pheonomenon in relation to symptoms experienced patients or pharmacological treatment-effects, may therefore benefit from the individualization of temperatures [11], [12], [25]. Also, we refrained from using noxious thermal stimuli for comparisons. This was partially for technical and logistic reasons but also due to the fact that we previously demonstrated that the TGI appears to be more unpleasant than painful [15] – perhaps rendering such stimuli less relevant. Nonetheless, the presently described system would allow for the presentation of temperatures as low as 5°C and as high as 47°C. Subject to a few modifications, the system could also accommodate additional water-baths – something which would permit more advanced study designs. Additionally, the TGI-system could be fitted with an array of commercially available MRI-compatible fibre-optic thermosensors. This would allow for an elaborate online monitoring during scanning. The exact temporal dynamics of the system could then be included as regressors in the data analysis. This would be important for event-related designs. For the present purposes, however, with epochs of 20 second long stimuli, the absence of such high temporal resolution is very unlikely to have had any relevant influence on our fMRI-analyses.

### Conclusion

We developed an MRI-compatible thermal grill illusion (TGI)-unit. To the best of our knowledge our study represents the first fMRI-investigation of the TGI. The behavioral results corroborate our previous findings [15] that the quality of the TGI-percept (for fixed cold and warm temperatures) appears to lie more along the affective-motivational than sensory-discriminative dimension. The right mid/anterior insular activation in response to the TGI (correlating with ratings of TGI-“unpleasantness”) is highly compatible with this region's proposed role in subjective feeling-states [29]. The imaging results constitute novel, direct evidence, for a thalamic involvement in the TGI. We propose the hypothesis that the special electrophysiological properties of thalamic neurons, relating to burst-activity, contribute to this finding. The TGI has been suggested as a model of percepts involved in neuropathic-pain, including cold-allodynia [5]. The thalamus is known to play an important role in such pain-pathologies and has been shown to be activated in response to evoked cold-allodynia in patients [20]. In sum, our results contribute to the understanding of the TGI-phenomenon per se. Future fMRI-studies comparing neuropathic pain with the TGI are now both possible and clearly warranted.

## Supporting Information

Video S1 A recording of a thermography session of the TGI-system is provided as a QuickTime-film. The thermography was carried out using a calibrated infrared (IR) camera with high thermal resolution (model 882, Testo AG, Lenzkirch, Germany). The video shows the presentation of 20 second long stimuli – i.e. warm (41.0±0.5°C) , TGI (18.0±0.5°C & 41.0±0.5°C) and cold (18.0±0.5°C) - separated by the neutral baseline (31.0±2°C).
(QT) Click here for additional data file.
